# Unveiling the Distribution Characteristics of Benzene-Based Pollutants in a Retired Industrial Park and Their Influence Factors: Soil Properties and Microbial Communities

**DOI:** 10.3390/toxics13090791

**Published:** 2025-09-17

**Authors:** Lei Wang, Weizhen Chen, Xuejun Tan, Li Xie

**Affiliations:** 1 Key Laboratory of Yangtze River Water Environment, Ministry of Education, College of Environmental Science and Engineering, Tongji University, 1239 Siping Road, Shanghai 200092, China; 2 Shanghai Municipal Engineering Design Institute (Group) Co., Ltd., Shanghai 200092, China; 3 Shanghai Institute of Pollution Control and Ecological Security, 1239 Siping Road, Shanghai 200092, China

**Keywords:** retired industrial parks, benzene-based pollutants, contamination characteristics, microbial distribution features

## Abstract

With the transformation of industrial enterprises in China, the relocation of numerous factories has led to the emergence of retired industrial parks with serious pollution. This study investigated the contamination of benzene-based pollutants (BBPs) in soil and their relationship with soil texture, physicochemical properties, and microbial communities at a former factory site in Shanghai. The results indicated that benzene and toluene were the main pollutants in the region, accounting for 25.7–36.1% and 7.6–10.2% of the total pollutants, respectively. The horizontal contamination distribution pattern of BBPs at different sampling points were clearly related to the functional zoning of the area. Sampling points close to workshops and bathrooms possessed higher contamination levels of BBPs than those close to warehouses and office buildings. With the increase in sampling depth, the gradually rising soil density and soil porosity ratio reduced the adsorption capacity of soil for BBPs, thereby promoting the volatilization and release of BBPs in deeper soil layers to a certain extent, resulting in a “shallow > deep” trend for the content of BBPs. The abundance of norank_f__norank__o_norank__c__Bathyarchaeia in the soil may be the main functional microorganisms affecting the distribution of BBPs. Styrene and chlorobenzene exhibited significant correlations with microbial communities, primarily involving bacteria (Desulfobacterium, Thermincola, and Trichlorobacter) and archaea (including norank_f_Nitrosopumilaceae, norank_f_norank_o_norank_c_Nitrososphaeria, and Methanocella). This study identifies and analyzes the BBP contamination characteristics in a typical retired industrial park in Shanghai, providing valuable references for risk assessment and microbial remediation of such contaminated areas.

## 1. Introduction

The Yangtze River Delta region (YRD) of China has a wide distribution of industrial enterprises and a rich variety of industry types, which are dominated by heavy industrial manufacturing and chemical enterprises. These factories use a large amount of industrial raw materials (such as chemical raw materials, ores, and petroleum) in their production processes, which can easily lead to organic pollution of the soil in the region. Polychlorinated biphenyl contamination (<0.1 to 130 ng/g dry weight) was found to be widespread in the YRD region and mainly concentrated in the soil of the shallow layer [1]. Additionally, key contaminants such as petroleum hydrocarbons, Di (2-ethylhexyl) phthalate, benzene, and ethylbenzene were detected in the soil of a retired industrial park in the YRD region [2]. With the transformation and progress of industrial enterprises in China, a large number of industrial enterprises and factories need to be closed down and relocated, and the retired industrial parks will be rezoned for residential, commercial, and public service facilities. However, the organic contamination of the soil may lead to a significant threat to human health during the development and use of these retired industrial parks. Therefore, it is necessary to systematically analyze and evaluate the spatial distribution characteristics of organic pollutants and pollution features in the soil of retired industrial parks in the YRD region.

Benzene based pollutants (BBPs), encompassing benzene, toluene, ethylbenzene, xylene, and other benzene derivatives, are among the primary contaminants in retired industrial sites. BBPs can pose severe threats to both ecosystems and human health by potentially causing leukemia, aplastic anemia, and cancer [3]. Due to their high toxicity, high mobility, and weak hydrophobicity, BBPs can migrate to deeper soil layers readily, resulting in certain degrees of groundwater contamination [4]. The migration and transformation of BBPs in soil are intimately correlated with soil texture and physicochemical properties. Zhang et al. [5] observed that when soil moisture is low, nearly all benzene in soil was adsorbed onto mineral surfaces. Wang et al. [6] reported that BBPs are adsorbed by clays during the migration from shallow soil to deep soil and subsequently released into groundwater due to the volatility of BBPs. On the other hand, low-permeability silt layers in deep soil may hinder the migration of benzene homologues, thereby reducing the transportation of BBPs from soil to groundwater [7]. Furthermore, the pH value of soil has been demonstrated to have a significant impact on the release of BBPs; this phenomenon may be attributed to the dissolution of BBPs by dissolved organic matters [8]. Nevertheless, few studies have explored the relationship between the spatial distribution characteristics of BBPs in soil and soil properties. Thus, it is imperative to identify the migration, transformation, and distribution characteristics of BBPs in soils at different depths by combining both soil texture and physicochemical properties.

The presence of BBPs in contaminated sites can also elicit alterations in microbial community structure and aggregation patterns [4,9]. Compared to uncontaminated soil, soil and groundwater contaminated by BBPs exhibit higher relative abundances of Helicobacter, Anaplasma, Firmicutes, Chloroflexi, and Deltaproteobacteria [9]. Ali et al. [10] observed the enrichment of *Pseudomonas* and *Rhodococcus* during biostimulation of benzene-contaminated soil, which suggested that dehydrogenase genes encoded by these microorganisms may be involved in the metabolism of benzene in contaminated soil. However, most of these studies were laboratory-scale experiments and lacked large-scale soil sampling and analysis of vast contaminated sites. In addition, numerous studies have explored the role of microorganisms in the degradation of BBPs. Xie et al. [11] identified Actinomycetes as an aerobic benzene-degrading strain. Sun and Sun [12] found that under methanogenic conditions, Proteobacteria (genus *Pseudomonas*) may be the dominant toluene-degrading bacteria, while bacteria associated with *Simplicispira* are also implicated in the anaerobic biodegradation of toluene. The addition of biostimulants such as *Pseudomonas*, *Rhodococcus*, *Noviherbaspirillum,* and *Nocardioides* can also enhance the biodegradation of benzene and benzopyrene under aerobic conditions [10]. The transmissible plasmids related to toluene, m-xylene, and p-xylene catabolism have also been detected in *Pseudomonas putida* [12]. Consequently, understanding the relationship between the BBP content in soil at different depths and the composition of microbial community structures will contribute to a better comprehension of how soil microbial communities respond to BBP pollution, providing theoretical support for the microbial remediation of BBP-contaminated soil.

Therefore, a retired industrial park in Shanghai in the YRD region was selected for investigation in this study. This study was based on the following hypotheses: (i) significant levels of BBP residues are present in the soils of the industrial park, and their spatial distribution and migration behavior are influenced by soil texture and physicochemical properties; (ii) BBP contamination exerts considerable effects on the soil’s microbial community structure and diversity, with specific microbial taxa showing significant correlations with BBP concentrations, indicating their potential utility as bioindicators. The specific objectives of the present study were to (i) investigate the soil BBPs pollution characteristics in the region and identify the migration patterns of BBPs in soil; (ii) explore the effects of soil texture and soil physicochemical properties on the distribution and transportation of BBPs; (iii) unveil the distributional characteristics of the microbial community in the soil contaminated by BBPs, then discuss the correlative relationship between BBP distribution and the microbial community. This study can provide a theoretical basis for the remediation of soil BBPs pollution in retired parks in the YRD region during the process of enterprise transformation, as well as for the subsequent planning and utilization of land in retired parks.

## 2. Materials and Methods

### 2.1. Collection and Determination of Soil Samples

**Figure 1:**
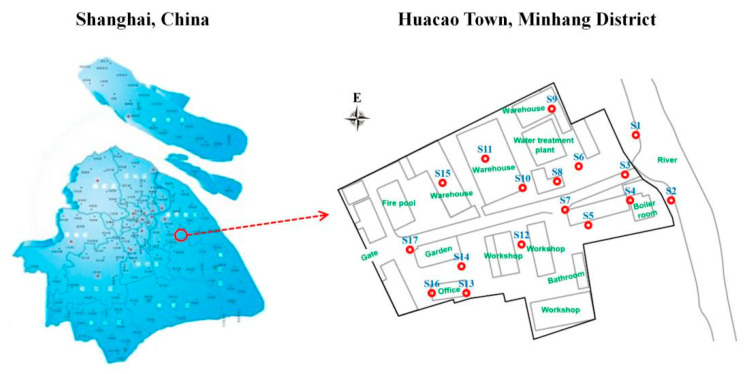
Schematic diagram of sampling areas and points.

In this study, the contaminated soil of a retired industrial park in Shanghai was selected as the sampling area, which is located in Huacao Town, Minhang District (121°33′ E, 31°22′ N). The sampling time was April to May 2021. The specific sampling locations are shown in Figure 1. A total of 17 sampling points (S1–S17) and 2 sampling depths (2.5 m and 7.0 m) were included in this study. Quality control measures were implemented during both sampling and laboratory handling to prevent the volatilization of BBPs. The specific procedures were as follows: Soil borehole drilling was conducted using a GeoProbe^®^ 7822DT via direct push technology. Approximately 2 cm of the outer surface of the soil core in the sampling tube was first scraped away. A non-disturbed sampler was then used to collect approximately 5 g of soil from the newly exposed inner surface of the core. The sample was immediately transferred into a 40 mL soil sampling vial pre-filled with 10 mL of analytical-grade methanol (Sinopharm Chemical Reagent Co. Ltd., Shanghai, China), taking care to avoid splashing of the methanol during transfer. After transfer, any soil adhered to the vial threads was quickly removed, and the cap was tightly sealed. The exterior of the vial was also cleaned of any adhered soil. All collected soil samples were immediately placed in a cooler with blue ice and delivered to the laboratory within 4 h for analysis.

Soil samples from three sampling points (S8, S12, S14) were selected based on the division of functional areas within the region for the determination of microorganisms, soil texture, and soil physicochemical properties. The soil samples for microbiological determinations were taken in 10 mL sterile plastic centrifuge tubes, then labeled accordingly and sent to the laboratory, where they were stored in a refrigerator at −80 °C for subsequent determinations. Benzene, toluene, ethylbenzene, xylene, styrene, and chlorobenzene from the soil were extracted based on US Environmental Protection Agency (US EPA) Methods 5035A, and the concentrations of contaminants were measured by Agilent 7890B-5977A GC/MS using US EPA Method 8260C; the detection limits for different BBPs were 0.0019 (benzene), 0.0013 (toluene), 0.0011 (styrene), 0.0012 (ethylbenzene, xylene, chlorobenzene). The texture of all soil samples is shown in Table 1.

### 2.2. Microbial Community Composition Analysis

Total microbial genomic DNA was extracted from soil samples using the FastDNA^®^ Spin Kit for Soil (MP Biomedicals, Irvine, CA, USA) according to the manufacturer’s instructions. The quality and concentration of the DNA were determined by 1.0% agarose gel electrophoresis and a NanoDrop2000 spectrophotometer (Thermo Scientific, Waltham, MA, USA). Samples were kept at −80 °C prior to further use. The hypervariable region V3–V4 of the bacterial 16S rRNA gene were amplified with primer pairs 338F (5′-ACTCCTACGGGAGGCAGCAG-3′) and 806R (5′-GGACTACHVGGGTWTCTAAT-3′) by T100 Thermal Cycler PCR thermocycler (BIO-RAD, Hercules, CA, USA). The primer pairs used for the determination of archaea were 524F10extF (TGYCAGCCGCCGCGGTAA) and Arch958RmodR (YCCGGCGTTGAVTCCAATT). The PCR reaction mixture including 4 μL 5 × Fast Pfu buffer, 2.5 mM dNTPs 2 μL, 0.8 μL each primer (5 μM), 0.4 μL Fast Pfu polymerase, 10 ng of template DNA, and ddH_2_O to a final volume of 20 µL. PCR amplification cycling conditions were as follows: initial denaturation at 95 °C for 180 s, followed by 27 cycles of denaturing at 95 °C for 30 s, annealing at 55 °C for 30 s and extension at 72 °C for 45 s, single extension at 72 °C for 10 min, and end at 10 °C. The PCR product was extracted from 2% agarose gel, purified using the PCR Clean-Up Kit (Yuhua, Shanghai, China) according to the manufacturer’s instructions, and quantified using Qubit 4.0 (Thermo Fisher Scientific, USA).

Data were analyzed using one-way analysis of variance (one-way ANOVA); statistical significance was set at *p* < 0.05. All data analyses of the microbial community were performed on the Majorbio BioCloud platform (https://cloud.majorbio.com). The figures were drawn using Origin 2023.

## 3. Results and Discussion

### 3.1. Influence of Soil Spatial Structure on BBP Distribution

**Figure 2:**
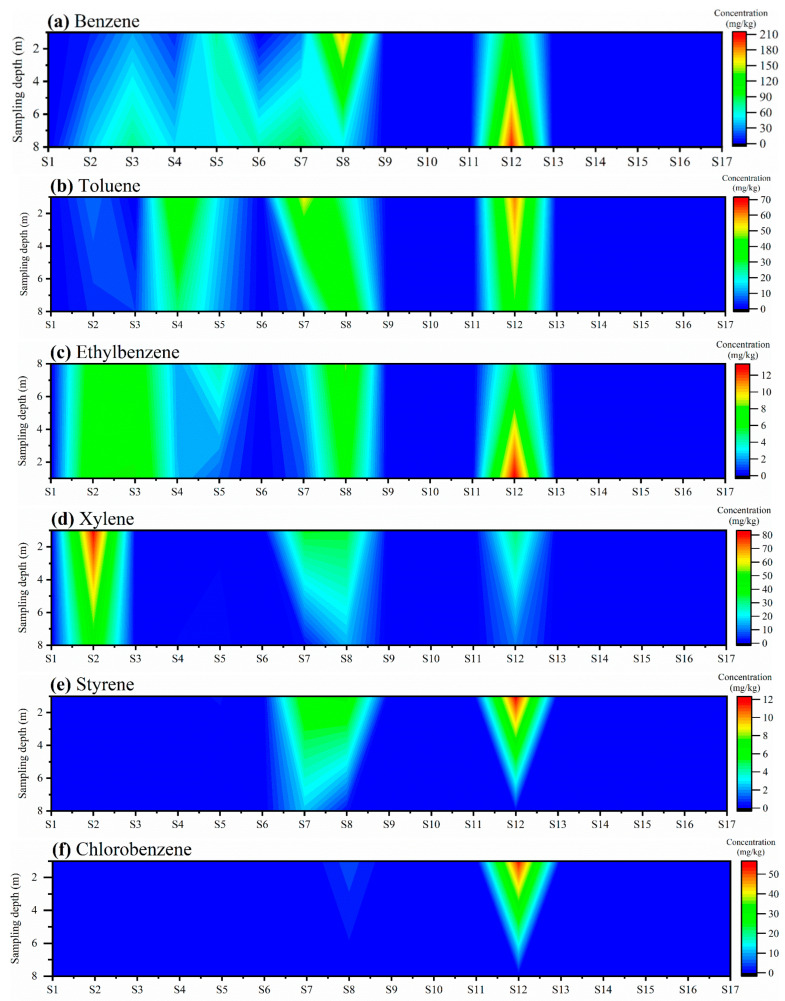
Content of benzene (**a**), toluene (**b**), ethylbenzene (**c**), xylene (**d**), styrene (**e**), chlorobenzene (**f**) in soil at different sampling depths.

Figure 2 demonstrates the distribution of BBPs in the soil of the retired industrial park. Among all the sampling points, nine sampling (S1–S8, S12) points were contaminated with BBPs to different degrees, while all the BBP concentrations at the other eight sampling points (S9–S11, S13–S17) were below the detection limit, and belonged to the “clean zone”. Benzene and toluene were the main pollutants in the region, with concentrations ranging from 0.02 to 202.00 mg/kg and 0.04 to 61.00 mg/kg (Figure 2a,b), accounting for 25.7 to 36.1% and 7.6 to 10.2% of the total pollutants, respectively. On the other hand, the pollution levels of ethylbenzene, xylene, styrene, and chlorobenzene in the soil of the region were relatively low; the concentrations of these BBPs only accounted for 1.8–2.2%, 2.1–7.7%, 0.3–1.9%, and 0.2–4.4% of the total pollutants, respectively (Figure 2c–f). This retired industrial park was once the site of a resin factory, where industrial raw materials (including styrene, benzene, toluene, chloroacetic acid, and dichloroethane) used in the production of resins were the main source of soil contamination, with various types of BBPs in the area. Sampling points (S9–S11, S13–S17) belonging to the “clean zone” were close to warehouses and office buildings, which are farther away from the initial sources of BBPs, so the contamination level of BBPs at these sampling points was slight. This phenomenon may have occurred due to the longer migration distances limiting the desorption of BBPs from the soil, which in turn resulted in differences in the contamination levels of BBPs between the different sampling sites [6].

Overall, the pollution level and vertical migration pattern of BBPs at different sampling points were clearly related to the functional zone classification of the region. The concentration of benzene at S6-2.5 was only 0.15 mg/kg, whereas the concentration of benzene at S6-7.0 reached 66.80 mg/kg. This phenomenon was probably due to the proximity of S6 to the water treatment plant, as the operation process of the water treatment plant might have affected the vertical migration of benzene at this sampling point. The proximity of S3–S5, S7–S8, and S12 to the workshops may also be the main reason for the high contamination levels of BBPs at these sampling points. Among all the soil samples, S12-7.0 had the highest concentration of benzene, reaching 202.00 mg/kg, which may be due to the proximity of S12 to the workshops, where pollutants generated during the production process could easily seep into the ground along with the domestic wastewater to cause serious benzene contamination. The “shallow < deep” pattern of benzene concentration at S7 may be due to similar reasons. In addition, toluene and ethylbenzene concentrations at S8 increased with the sampling depth from 22.70 mg/kg and 6.32 mg/kg to 44.40 mg/kg and 8.48 mg/kg, respectively; whereas at S12, toluene and ethylbenzene concentrations showed the opposite trend to that of S8, decreasing from 61.00 mg/kg and 13.00 mg/kg to 43.30 mg/kg and 5.35 mg/kg, respectively. This discrepancy can be explained by the difference in soil texture; the presence of some silt layers in deeper soils may hinder the migration of BBPs [7]. There was infiltration of xylene, ethylbenzene, and chlorobenzene at S12. This may be due to the high water solubility of these three pollutants [12]. On the other hand, at the 2.5 m depth, the concentrations of BBPs at sampling sites with relatively severe contamination (S2–S5, S7, S8, S12) were 237.26 to 2099.31 times higher than those at S1, S6, and S14 (*p* < 0.05). Similarly, at the 7.0 m depth, the BBP concentrations at more contaminated sites (S2–S5, S7, S8, S12) exceeded those at S1, S6, and S14 by factors ranging from 2.54 to 1044.97 (*p* < 0.05). These results further demonstrate that the historical functional zoning of the sampling areas, combined with the subsurface soil stratification, exerted a substantial influence on both the extent of BBP contamination and its vertical migration behavior.

### 3.2. Influence of Soil Physicochemical Properties on the Distribution of BBPs

**Figure 3:**
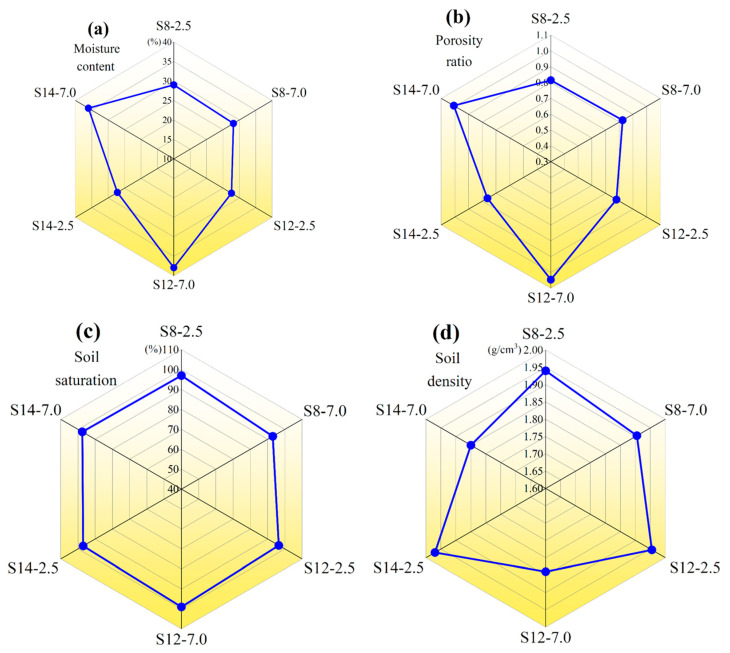
Moisture content (**a**), porosity ratio (**b**), saturation (**c**), and density (**d**) of soil samples. S8, S12, and S14 represent three distinct representative sampling points, while the values 2.5 and 7.0 denote different sampling depths (in meters).

Figure 3 depicts the variation in soil physicochemical properties at different sampling points. As shown in Figure 3a,b, the soil moisture content and soil porosity ratio of S12-7.0 were 37.9% and 1.0, respectively, which were 37.3% and 25.0% higher than those of S12-2.5. The higher water content and porosity ratio imply that more soil pore channels will be occupied by water, which is not favorable for benzene volatilization [5,13,14]. Therefore, the benzene content at S12-7.0 was the highest among all treatments. In addition, the soil saturation at S12-7.0 was 99.0, which was the highest among all sampling points (Figure 3c), explaining why the BBP contamination level at S12 was higher than those at the other sampling points. The higher soil saturation may facilitate the uptake of benzene at the air–solid interface in the soil [5]. Overall, soil porosity ratio and soil density increased with soil depth (Figure 3b,d). This phenomenon implies a decrease in soil adsorption of benzene and to some extent promotes the volatilization and release of benzene pollutants from the deeper layers of soil [15,16]. Therefore, the concentrations of most BBPs at the three sampling points in this study decreased with increasing sampling depth.

Based on EN ISO 14688-2 (2018), the soil textures at different depths in the study area were classified [17]. The soil textures of both S8-2.5 and S8-7.0 are classified as silty clay, so it is easy for BBPs to diffuse and migrate (Table 1). Consequently, a general trend of “shallow > deep” was observed for BBPs in S8. Notably, at S8, the concentration of toluene varied inversely to that of other BBPs within the same soil texture, which could possibly be attributed to competitive sorption between benzene and toluene in the soil. Specifically, the benzene concentration at S12-2.5 was 106.00 mg/kg, while it increased to 202.00 mg/kg at S12-7.0. One possible reason for this phenomenon is that S12 is close to the bathroom, and the leakage of nearby domestic wastewater accelerates the vertical migration of BBPs in the soil layer. The soil textures of S12-2.5 and S12-7.0 are silty clay and muddy clay, respectively. Hence, BBPs rapidly migrate downwards with the leaching of domestic sewage and subsequently adsorb onto the muddy layer in the deep layer of soil [7]. This explains the substantial increase in benzene concentration at S12-7.0 compared to S12-2.5. Notably, the concentrations of toluene and chlorobenzene are similar at S12-2.5 but vastly differ at S12-7.0. This phenomenon may stem from the higher solubility of chlorobenzenethan compared to toluene, enabling stronger migration and diffusion of chlorobenzene within the muddy layer [18]. Consequently, the groundwater at S12-7.0 is likely to be severely contaminated by chlorobenzene. The content of each BBP at the S14 sampling point was relatively lower than at S8 and S12, so the effect of the differences in soil texture on the transportation and transformation of BBPs was not significant.

### 3.3. Microbial Communities in the Soil of Different Sampling Points

**Figure 4:**
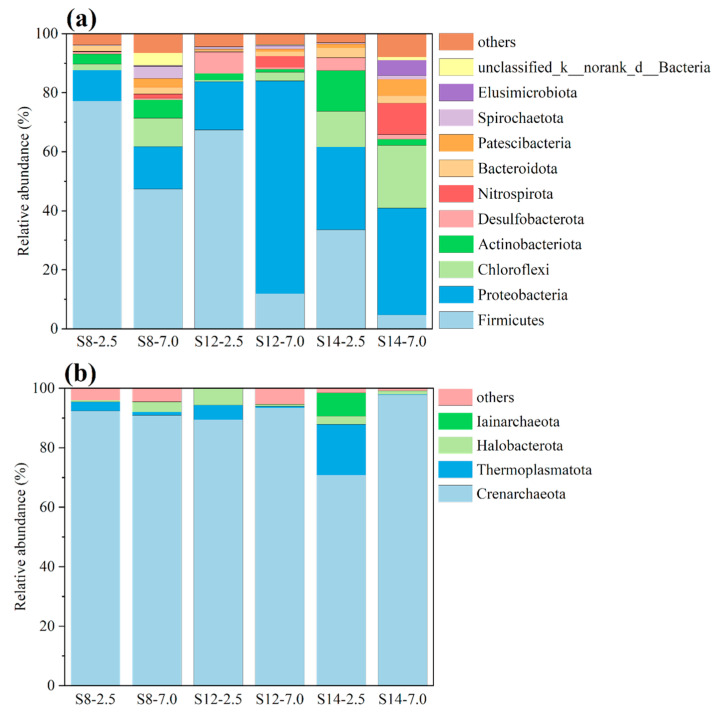
Microorganisms at the phylum level in soil samples at different sampling depths, including bacteria (**a**) and archaea (**b**). S8, S12 and S14 represent three distinct representative sampling points, while the values 2.5 and 7.0 denote different sampling depths (in m).

The relationship between alterations in microbial community structure and the distribution pattern of BBPs at three sampling points was elucidated through comparative analysis. This analysis focused on the differences in microbial communities between S8 and S12, characterized by higher contamination levels, and S14, located in a clean area. Generally, the microbial diversity indexes at different sampling points did not show a clear pattern of change (Table S1), due to the combined pollution of many types of BBPs. Figure 4a shows the composition of the community structure of the bacteria in the soil at the phylum level. Firmicutes (4.7–77.2%) and Proteobacteria (10.5–72.1%) were the dominant microorganisms at these sampling points. Similar results were also found in the study by Berlendis et al. [19]; most of the microorganisms in an area contaminated by benzene, toluene, ethylbenzene, and xylene belonged to the Firmicutes and Proteobacteria families. At the same sampling depth, the relative abundance of Firmicutes was higher in S8 and S12 than in S14, which may be related to the fact that BBP contamination was more serious in S8 and S12 than in S14. The relative abundance of Proteobacteria in S12-7.0 was much higher than in the other sampling points, which may be the main reason for the change in toluene in the “shallow > deep” pattern for S12. This was also confirmed by Sun et al. [12]; the bacteria belonging to the Proteobacteria family may have functions related to toluene degradation. The community of archaea was dominated by Crenarchaeota, with relative abundance ranging from 70.9 to 97.8% (Figure 4b). Crenarchaeota were also found to be associated with diffusive effects and the concentration of the release of BBPs in soil in previous studies [20,21]. Therefore, Crenarchaeota may be the main archaeal community that affected the changes and distribution of BBPs in the abandoned industrial park. At S12 and S14, the relative abundance of Acidobacteriota increased as the soil texture transitioned from silty clay to silty clay with muddy characteristics. Previous studies have also identified soil properties as among the most influential predictors of Acidobacterial abundance [22]. The lower hydraulic conductivity in the deep muddy clay layers promotes the formation of anoxic conditions, which in turn provides an ideal niche for Acidobacteriota. This texture-driven distribution pattern suggests that Acidobacteriota may play a key role in the deep migration and transformation of BBPs.

**Figure 5:**
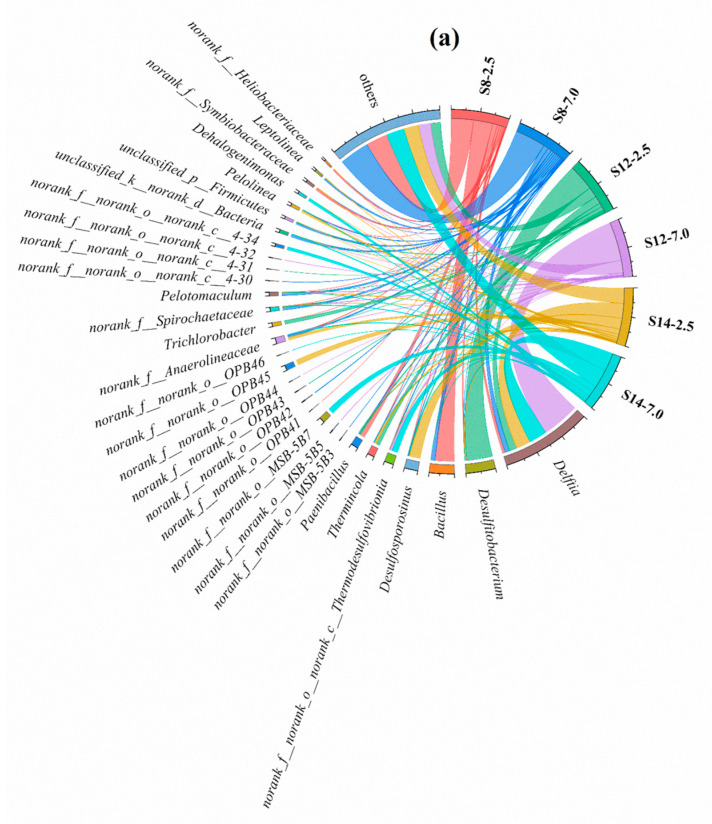

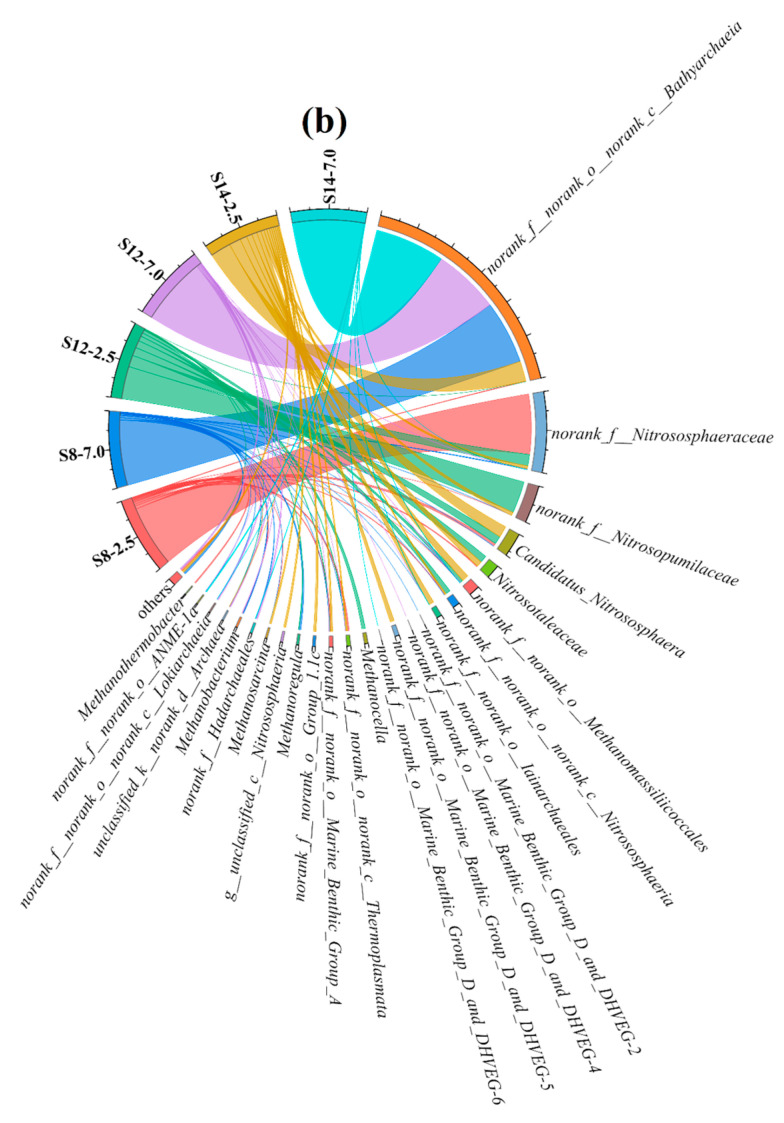
Microorganisms at the genus level in soil samples at different sampling depths, including bacteria (**a**) and archaea (**b**). S8, S12, and S14 represent three distinct representative sampling points, while the values 2.5 and 7.0 denote different sampling depths (in m).

The bacterial community compositions at the genus level of all sampling points are shown in Figure 5a. *Delftia* (4.5–69.4%), *Desulfitobacterium* (0.01–47.6%), and *Bacillus* (0.2–34.8%) were the dominant microorganisms. The relative abundance of *Delftia* and *Desulfitobacterium* showed a “shallow < deep” trend at all sampling points. *Desulfitobacterium* was found to degrade hydrocarbons in the study of Ramos et al. [21], and a study by Vásquez et al. [23] found that *Delftia* can degrade benzene. Therefore, the concentration of benzene at S8-7.0 is lower than that of S8-2.5. The reason for the “shallow < deep” pattern of benzene concentration at S12 may be the large degree of enrichment of benzene at S12-7.0 caused by the presence of a larger silt layer. At S14, the degree of BBP contamination was slight, thus the changes in relative abundance for *Delftia* and *Desulfitobacterium* at different depths had few influences on the concentration of BBPs. *Bacillus* was mainly enriched at S8, and the relative abundance of *Bacillus* at S8-2.5 (34.8%) was higher than that of S8-7.0 (7.0%), which led to the “shallow > deep” concentration of benzene and xylene at S8. This phenomenon may have been caused by the fact that some microorganisms belonging to *Bacillus* (e.g., *Bacillus* subtilis DM-04) have the function of degrading benzene and xylene [24].

Figure 5b shows the distribution of the archaea community in the soil at the genus level. *norank_f__norank__o_norank__c__Bathyarchaeia* (0.8–97.1%), *norank_f_Nitrososphaeracese* (0.2–83.5%), and *norank_f_ Nitrosopumilaceae* (4.5–44.5%) were the dominant microorganisms. *norank__f__norank__o_norank__c__Bathyarchaeia* was mainly distributed in the deep layer of soil at S14-7.0. It had a higher relative abundance than that of at S8 and S12, which may be due to the difference in BBP contamination levels between these sampling points. As demonstrated in previous studies, *Bathyarchaeia* has been found to be highly metabolized under anoxic conditions and to be capable of dominating petroleum degradation [25,26]. It can therefore be concluded that the variation in *norank_f_norank_o_norank_c__Bathyarchaeia* in soil may be the primary reason for the differences in the distribution pattern of BBPs at different sampling points. The *Noran_f_Nitrososphaeracese* may be a characteristic microorganism associated with benzene. The relative abundance of *norank_f_Nitrosopumilaceae* at S12-2.5 reached 44.5%, while the relative abundance of *norank_f_Nitrosopumilaceae* at the other sampling points was much lower than S12, which may be caused by the higher toluene concentration at S12-2.5. The relative abundance of *Noran_f_Nitrososphaeracese* at S8-2.5 reached 83.5%, which is much higher than other sampling points. This may be related to the higher benzene concentration at S8-2.5. Overall, at S8, S12, and S14, both BBP concentrations and the relative abundance of *norank_f__Nitrososphaeraceae* exhibited a consistent “shallow > deep” vertical pattern, indicating significant spatial co-occurrence between this group of ammonia-oxidizing archaea and BBP contamination. *Nitrososphaeraceae* play a crucial role in soil nitrogen cycling, particularly in ammonia oxidation [27]. The nitrite produced via *Nitrososphaeraceae*-mediated ammonia oxidation can serve as a key electron acceptor for heterotrophic degraders capable of denitrification, thereby indirectly driving the anaerobic degradation of BBPs through microbial interactions.

### 3.4. Correlation Between Microorganisms at the Genus Level and Benzene Pollutants

**Figure 6:**
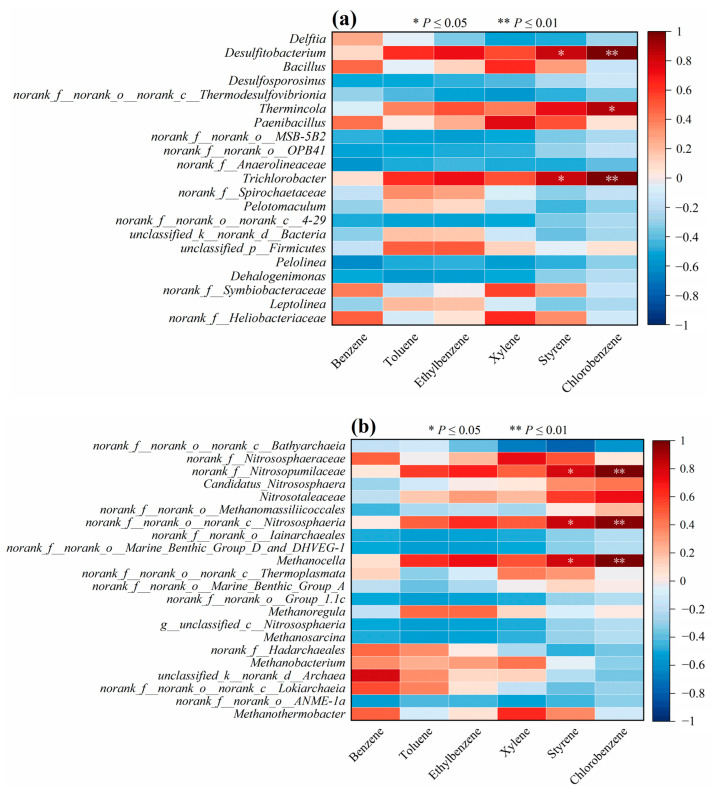
Correlation between bacteria (**a**) and archaea (**b**) at the genus level and BBPs.

In order to further investigate the relationship between microbial composition and the level of soil contamination, a correlation analysis between different types of microorganisms at the genus level and the BBP content in the soil was conducted. Significant positive correlations (*p* < 0.05) were found between both *Desulfitobacterium* and *Trichlorobacter* and styrene and chlorobenzene (as shown in Figure 6a). *Desulfitobacterium* was found to present in BBP-contaminated soil and could degrade hydrocarbons under anaerobic conditions [21]. Significant positive correlations were found between *Thermincola* and chlorobenzene (*p* < 0.05), which means the changes in the relative abundance of *Trichlorobacter* may also be related to the transportation and transformation of chlorobenzene. *Trichlorobacter* is an electric bacterium. The tracking and analysis of the specific gene abundance of *Thermincola* and its growth phase in the remediation project will be beneficial for carrying out biodegradation studies of BBPs [28,29].

For the archaea communities at the genus level, *norank__f__Nitrosopumilaceae*, *norank__f__norank__o___norank__c__Nitrososphaeria*, and *Methanocella* all showed significant positive correlations with styrene and chlorobenzene (*p* < 0.05) (Figure 6b). Li et al. [30] found that *Nitrosopumilaceae* existed in polycyclic-aromatic-hydrocarbon-contaminated soil. Polycyclic aromatic hydrocarbons and BBPs have similar physicochemical properties, which may be the reason for the significant correlation observed between these microorganisms and BBPs in this study. In addition, changes in the relative abundance of *Nitrosopumilaceae* may also affect the migration and transformation of polycyclic aromatic hydrocarbons and BBPs. Li et al. [21] identified *Nitrososphaeria* as a soil proto-microorganism with a phenanthrene-degrading function. *Nitrosopumilaceae* and *Nitrososphaeria* also participate in the nitrogen cycling process in the soil, and the analysis of the distribution of these two archaea communities may be beneficial to further investigation of the transportation and transformation of BBPs in contaminated soil [31]. The archaea Methanocella was discovered in paddy soil contaminated with phenol. The biodegradation function of Methanocella was explored through the addition of lactic acid. It is hypothesized that this archaeon has a direct relationship with the biodegradation process of BBP-containing compounds in soil under anaerobic conditions. *Methanocella* was found in phenol-contaminated paddy soil, and it is likely that this archaeon has a direct relationship with the biodegradation process of BBP-containing compounds in soil under anaerobic conditions [32].

**Figure 7:**
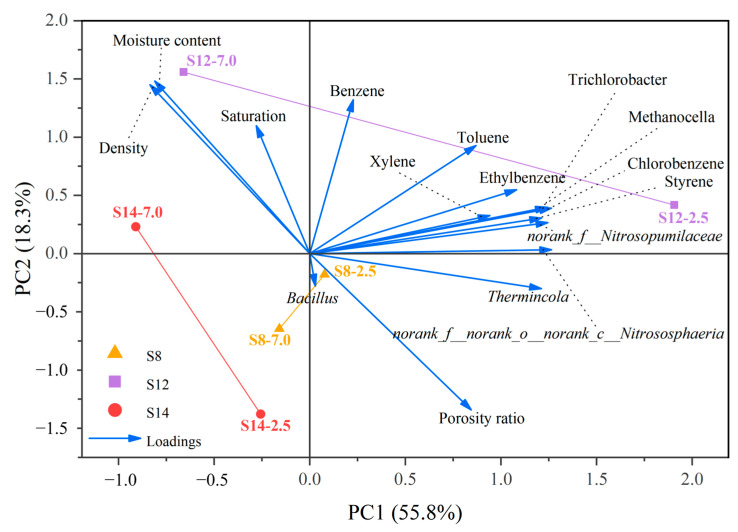
Principal component analysis of BBP concentrations, soil physicochemical properties, and microorganisms showing significant correlations with BBPs. S8, S12, and S14 represent three distinct representative sampling points, while the values 2.5 and 7.0 denote different sampling depths (in m).

A principal component analysis was further conducted to investigate the relationships among soil BBP concentrations, physicochemical properties, and microorganisms significantly correlated with BBPs (Figure 7). The contributions of PC1 and PC2 were 55.8% and 18.3%, respectively, collectively explaining 74.1% of the total variance. This indicates that the sample distribution shown in the plot accurately captures the true underlying structure of the original high-dimensional dataset. The considerable spatial separation among S8, S12, and S14 in the score plot reliably reflects fundamental differences in their soil properties, likely attributable to their distinct historical functional zoning. S14 is located on the left side of the negative PC1 axis, while both S12 and S8 are situated on the positive PC1 side but are clearly distinguished from each other along the PC2 axis. This complete spatial segregation suggests that the dominant factors driving soil properties at each site are fundamentally different.

Specifically, both S8-2.5 and S12-2.5 are located in the positive PCI region and align closely with the vectors of BBPs and key functional microorganisms (e.g., *Thermincola*, *Methanocella*), indicating that site-specific microbial responses—particularly the enrichment of anaerobic degraders—likely play a more critical role in shaping BBP contamination than soil physicochemical properties do at these locations. In contrast, S14 is positioned along the negative PC1 axis and correlates strongly with variables such as moisture content, density, and saturation, suggesting that intrinsic physical structure and hydrological conditions are the primary reasons for its significantly lower contamination level compared to S8 and S12.

### 3.5. Risk Assessment and Environmental Implication

A risk assessment of BBPs in the decommissioned industrial park was conducted based on GB 36600-2018 [33]. The results indicated that the maximum detected concentrations of benzene and ethylbenzene (202.00 mg/kg, 13.00 mg/kg) exceeded the screening values (1.00 mg/kg, 7.20 mg/kg) (Table S2). Further human health risk assessment was performed for BBP concentrations in the area using the model and parameters recommended in the Technical Guidelines for Risk Assessment of Soil Contamination in Development Land (HJ25.3-2019). The carcinogenic risk and non-carcinogenic hazard quotient of BBPs for exposed receptors were calculated (Table S2). A risk level is considered unacceptable for potential5 exposed populations (adults and children) if the carcinogenic risk of a pollutant exceeds 10^−6^ or its non-carcinogenic hazard quotient exceeds 1. The results revealed that benzene and ethylbenzene posed significantly higher carcinogenic risks than other types of BBPs, while toluene exhibited the highest non-carcinogenic hazard quotient (1.594). These findings suggest that toluene and ethylbenzene are the dominant risk drivers in the area and should be prioritized for control and remediation. In contrast, the risks posed by toluene, xylene, styrene, and chlorobenzene are within acceptable limits, warranting long-term monitoring rather than immediate high-cost remediation measures.

This study identified the distribution characteristics of BBP contamination in a retired industrial site and further evaluated the migration, transformation, and environmental risks of BBPs based on soil properties, physicochemical indicators, and microbial community composition. Overall, the findings provide important guidance for designing bioremediation strategies targeting BBP pollution in similar retired industrial environments. Specifically, microorganisms significantly correlated with BBPs (e.g., *Desulfitobacterium*, *Thermincola*) may serve as candidate strains for bioaugmentation, enabling in situ or ex situ remediation of contaminated soils. Tailoring remediation strategies based on soil physicochemical indicators (e.g., moisture content, porosity, saturation, bulk density) and layer-specific properties (e.g., silty clay or silty clay with muddy characteristics) could further enhance treatment efficiency.

As this study was conducted as a field investigation at an actual engineering scale, the sample size and biological replication were relatively limited. In addition, molecular approaches such as metagenomics were not incorporated to deeply resolve functional genes and metabolic pathways. Future work will focus on expanding biological replicates and integrating metagenomic and metatranscriptomic analyses to more comprehensively elucidate the microbial molecular mechanisms underlying BBP degradation. These efforts will provide a theoretical basis and data support for developing efficient and practical bioremediation agents. Despite these limitations, this study offers initial insights into the distribution patterns of BBPs, key environmental drivers, and microbial responses at the field scale, thereby providing a scientific foundation for subsequent remediation practices.

## 4. Conclusions

This study investigated the contamination of BBPs for a retired industrial park in Shanghai and analyzed the influence of soil physicochemical properties, soil texture, and microbial communities on the distribution of BBPs in the soil. The results demonstrated that among all the sampling points, nine sampling points were contaminated with BBPs to different degrees, while the BBP concentrations at the other eight sampling points were below the detection limit. Benzene and toluene emerged as the primary pollutants in the region, accounting for 25.7–36.1% and 7.6–10.2% of the total pollutants, respectively. The contamination level and vertical migration pattern of BBPs at different sampling points were clearly related to the functional zoning of the area. Sampling points close to workshops possessed higher contamination levels of BBPs than those close to warehouses and office buildings. At S12, most of the BBPs rapidly penetrated the surface layer of silty clay and migrated to the deep layers of soil due to the leaching effect of domestic sewage, and then adsorbed in the deep soil silt layer, resulting in the changed trend of benzene at S12 with the pattern of “deep > shallow”. The decrease in the concentrations of most BBPs with increasing sampling depth at all sampling points were likely attributed to the enhanced soil density and soil porosity ratio, which diminish the adsorption capacity of soil for BBPs. Furthermore, styrene and chlorobenzene exhibited significant correlations with microbial communities, primarily involving bacteria (*Desulfobacterium*, *Thermincola*, and *Trichlorobacter*) and archaea (*norank_f_Nitrosopumilaceae*, *norank_f_norank_o_norank_c_Nitrososphaeria*, and *Methanocella*). The presence of these microorganisms may be attributed to their inherent degradation capabilities towards BBPs.

The results of risk assessment suggest that toluene and ethylbenzene are the dominant risk drivers in the area and should be prioritized for control and remediation. In contrast, the risks posed by toluene, xylene, styrene, and chlorobenzene are within acceptable limits, warranting long-term monitoring rather than immediate high-cost remediation measures. In conclusion, this study provides systematic and comprehensive theoretical support for the remediation and risk assessment of BBPs contaminated areas brought by the relocation of factories.

## Figures and Tables

**Table 1:** The texture of soil samples in different sampling points.

Sampling Site	Soil Texture
S8-2.5	Silty clay
S8-7.0	Silty clay
S12-2.5	Silty clay with muddy characteristics
S12-7.0	Silty clay
S14-2.5	Silty clay
S14-7.0	Silty clay with muddy characteristics

## Data Availability

The data for this article can be obtained by contacting the corresponding author.

## Supplementary Materials

The following supporting information can be downloaded at: https://www.mdpi.com/article/10.3390/toxics13090791/s1, Table S1. The alpha diversity index of bacteria and archaea at different sampling points. Table S2. Risk Assessment of BBPs based on soil contamination risk guideline values.
