# Supplementary material for: Unveiling the Distribution Characteristics of Benzene-Based Pollutants in a Retired Industrial Park and Their Influence Factors: Soil Properties and Microbial Communities
Source: Toxics. 2025 Sep 17;13(9):791. doi: 10.3390/toxics13090791 (PMC12474079; doi:10.3390/toxics13090791)
Supplement: Supplementary file 1 [file toxics-13-00791-s001.zip › toxics-3859430-supplementary.pdf]

Table S1. The alpha diversity index of bacteria and archaea at different sampling points

	**Alpha index**	S8-2.5	S8-7.0	S12-2.5	S12-7.0	S14-2.5	S14-7.0
**Bacteria**	Chao	192	473	247	221	185	270
	Shannon	3.20	4.60	2.44	1.96	2.95	3.16
	Simpson	0.14	0.03	0.26	0.48	0.13	0.14
**Archaea**	Chao	21	35	13	21	21	15
	Shannon	0.85	0.65	1.71	0.45	2.38	0.18
	Simpson	0.70	0.79	0.25	0.86	0.13	0.94

Table S2. Risk Assessment of BBPs based on soil contamination risk guideline values

**BBPs** (mg/kg)	Maximum detected concentration	Screening value (mg/kg)	Carcinogenic risk	Non-carcinogenic hazard quotient
Benzene	202.00	1.00	1.55877E-05	1.594
Toluene	61.00	1200.00	-	0.023
Ethylbenzene	13.00	7.20	2.04302E-07	0.004
Xylene	35.10	385.00	-	0.008
Styrene	11.90	1290.00	-	0.002
Chlorobenzene	53.60	68.00	-	0.090
